# Isolation and molecular characterization of multidrug-resistant Gram-negative bacteria from imported flamingos in Japan

**DOI:** 10.1186/1751-0147-51-46

**Published:** 2009-11-24

**Authors:** Maiko Sato, Ashraf M Ahmed, Ayako Noda, Hitoshi Watanabe, Yukio Fukumoto, Tadashi Shimamoto

**Affiliations:** 1 Laboratory of Food Microbiology and Hygiene, Graduate School of Biosphere Science, Hiroshima University, Higashi-Hiroshima 739-8528, Japan; 2 Department of Bacteriology, Mycology and Immunology, Faculty of Veterinary Medicine, Kafr El-Sheikh University, Kafr El-Sheikh 33516, Egypt; 3 Hiroshima City Asa Zoological Park, Asa-cho Asakita-ku, Hiroshima 731-3355, Japan

## Abstract

Imported animals, especially those from developing countries, may constitute a potential hazard to native animals and to public health. In this study, a new flock of lesser flamingos imported from Tanzania to Hiroshima Zoological Park were screened for multidrug-resistant Gram-negative bacteria, integrons and antimicrobial resistance genes. Thirty-seven Gram-negative bacterial isolates were obtained from the flamingos. Seven isolates (18.9%) showed multidrug resistance phenotypes, the most common being against: ampicillin, streptomycin, tetracycline, trimethoprim/sulfamethoxazole and nalidixic acid. Molecular analyses identified class 1 and class 2 integrons, β-lactamase-encoding genes, *bla*_TEM-1 _and *bla*_CTX-M-2 _and the plasmid-mediated quinolone resistance genes, *qnrS *and *qnrB*. This study highlights the role of animal importation in the dissemination of multidrug-resistant bacteria, integrons and antimicrobial resistance genes from one country to another.

## Findings

The introduction of an exotic pathogen via imported animals has the potential to impact on all native animal species and on human health. Although all imported animals have to be certified as healthy and free from infectious and contagious diseases, these animals can act as carriers and reservoirs for many pathogenic and non-pathogenic microorganisms [1]. Furthermore, many animals undergo extreme migration patterns and this, combined with the translocation of animals, especially wild and zoo animals, may thus result in the spread of imported zoonoses from one country to another [2].

Currently, essential and life-saving antibiotics are becoming less effective, with fewer alternatives available for treatment [3,4]. The genes for resistance traits can be transferred among bacteria of different taxonomic and ecological groups. The increasing occurrence of antibiotic-resistant microorganisms has fueled interest in the genetics and mechanisms of resistance evolved by bacteria to counteract the effect of antimicrobial agents. The fact that resistance genes do not respect phylogenetic, ecological or geographical boundaries implies that antimicrobial use and the resulting resistance in one ecological niche may have consequences for the resistance situation in another niche [1].

The scientific data on the role of imported animals in the spread of multidrug-resistant (MDR) bacteria and resistance genes from one country to another is very limited. Also, little is known about the molecular basis of resistance in MDR bacteria isolated from imported animals, therefore, this study was conducted to detect and characterize the antimicrobial resistance genes in MDR Gram-negative bacteria isolated from an imported flock of flamingos at Hiroshima Zoological Park, Japan.

The imported flock of lesser flamingos (*Phoeniconaias minor*) consists of 20 birds. They were free-living wild birds in Tanzania. The lesser flamingo (*Phoeniconaias minor*) is the most abundant of all flamingos. It breeds mainly in the Rift Valley lakes of East Africa in Ethiopia, Kenya and Tanzania and covers a much broader geographical range during the non-breeding season (including sub-Saharan regions, the Arabian peninsula and parts of Asia) [5]. They were transported by airplane through the following pathway: Tanzania-Netherland-Osaka (Japan) airports. Then, they were overland transported from Osaka to Asa Zoological Park, Hiroshima, Japan. This flock arrived at Hiroshima City Asa Zoological Park, Hiroshima, Japan on 28^th ^December 2006 and sampling was made on the next morning. The imported flamingos were kept in the quarantine space and isolated from other animals. All feed, mat, manure, etc. were completely isolated from other areas. No antimicrobials were given to the flamingos before sampling. A total of 14 swabs (8 fecal and 6 bathing water swabs) were randomly taken from a recently imported flock of lesser flamingo from Tanzania. The fecal swabs were taken instead of cloacal swabs to avoid the direct handling of the birds. The swabs were immersed in buffered peptone water and dispatched immediately at 4°C to the laboratory where bacterial isolation was performed within 24 h. The isolates were isolated and identified using conventional techniques [6] and results were confirmed, where necessary, by the API 20E system (BioMérieux, Marcy-l'Étoile, France). A total of 37 Gram-negative bacterial isolates were recovered from lesser flamingos. Biochemical identification showed that the most prevalent species were *Escherichia coli *18 (48.6%), followed by *Pseudomonas aeruginosa *4 (10.8%), *Edwardsiella tarda *3 (8.1%), *Proteus mirabilis *3 (8.1%), *Citrobacter koseri *2 (5.4%), and single isolates (2.7%) of *Citrobacter youngae, Enterobacter cloacae, Klebsiella pneumoniae*, *Klebsiella oxytoca*, *Morganella morganii*, *Proteus vulgaris*, *Salmonella enterica *subsp. *arizonae*.

The antimicrobial sensitivity phenotypes of recovered bacteria were determined using a Kirby-Bauer disk diffusion assay according to the standards and interpretive criteria described by CLSI [7]. The antimicrobial diffusion disk tests showed that 7 of the bacterial isolates (18.9%) (4 isolates of *E. coli *and a single isolate of *C. youngae, E. cloacae *and *S. enterica *subsp.*arizonae*) showed multidrug resistance phenotypes to three or more antimicrobial agents. The most common resistance phenotypes were against: ampicillin, streptomycin, tetracycline, trimethoprim/sulfamethoxazole and nalidixic acid. Similar resistance phenotypes have been previously recorded for strains of *E. coli *isolated from wild animals in Portugal, from free-living Canada geese in Georgia and North Carolina (USA) and from black-headed gulls in the Czech Republic [8-10]. More recently, we have reported multiple resistance phenotypes in many Gram-negative bacteria isolated from zoo animals at Hiroshima Zoological Park [11]. Of note, Gram-negative bacteria with high level of resistance especially to ampicillin and trimethoprim/sulfamethoxazole were previously isolated from urinary tract infections in pregnant women from Tanzania [12]. In addition, from the public health point of view, three isolates showed resistance phenotypes to several clinically-important extended-spectrum β-lactam antibiotics such as cefotaxime, cefpodoxime, ceftriaxone and aztreonam [4]. Previously, in Japan, a study showed that vancomycin-resistant enterococci (VRE) isolated from imported chickens could contribute to the colonization of VRE in the intestines of healthy Japanese people who had ingested uncooked chickens [13]. Also, in Japan, pet birds imported from tropical countries were found to carry MDR bacteria (they are frequently treated with antibiotics) [14]. Therefore, the transmission of non-pathogenic multidrug-resistant (MDR) bacteria by imported animals is also important because they can serve as a reservoir for resistance genes that may be transferred to pathogens [1].

Integrons are genetic elements that can integrate gene cassettes, usually antibiotic resistance genes, by site-specific recombination [15]. The class 1 integron primers, 5'-CS and 3'-CS, which amplify the region between the 5'-conserved segment (5'-CS) and 3'-CS of class 1 integrons, were used as previously described (Table 1) [11]. Also, for the detection of class 2 integrons, PCR was performed with the primer pair, hep74 and hep51, specific to the conserved regions of the class 2 integrons, as described previously (Table 1) [11]. PCR-screening results detected class 1 integrons in two isolates of *E. coli *and one isolate of *S. enterica *subsp.*arizonae *(Table 2). Although, *S. enterica *subsp.*arizonae *seldom infects humans, a fatal case of gastroenteritis due to *S. enterica *subsp.*arizonae *has been reported in a 3-month-old child presented to the Emergency Unit in New Delhi, India [16]. DNA-sequencing results for the inserted gene cassettes within class 1 integrons identified only one gene cassette of dihydrofolate reductase, *dfrA7*, which confers resistance to trimethoprim. It is noteworthy that class 1 integrons with different gene cassettes (*dfrA1*, *dfrA5*, *dfrA12*, *dfrA15 dfrA17 *and *aadA1*, *aadA2*, *aadA5*) were previously reported from zoo animals at Hiroshima Zoological Park [11]. Class 1 integrons harboring *aadA1 *have been previously identified in *E. coli *isolated from free-living Canada geese in Georgia and North Carolina (USA) and black-headed gulls in the Czech Republic [8,10]. On the other hand, class 2 integron with a fragment size of 1581 bp was identified in a single isolate of *E. coli*. This class 2 integron was identical to that of Tn*1826*, and contained *sat2 *and *aadA1 *gene cassettes (Table 2) [17]. Notably, our group recently identified a class 2 integron carrying the three classical gene cassettes, *dfrA1*, *sat2 *and *aadA1*, in three *E. coli *strains isolated from wild birds, horned owl and white pelican housed at Hiroshima Zoological Park, Japan [11].

**Table 1:** Primers used in this study

Primer	Sequence (5' to 3')	Target	Reference
Integrons			

5'-CS 3'-CS	GGCATCCAAGCAGCAAG AAGCAGACTTGACCTGA	Class 1 integron	11

hep74 hep51	CGGGATCCCGGACGGCATGCACGATTTGTA GATGCCATCGCAAGTACGAG	Class 2 integron	11

β-Lactamases			

TEM-F TEM-R	ATAAAATTCTTGAAGACGAAA GACAGTTACCAATGCTTAATC	*bla*_TEM_	11

SHV-F SHV-R	TT ATCTCCCTGTTAGCCACC GATTTGCTGATTTCGCTCGG	*bla*_SHV_	11

OXA-F OXA-R	TCAACTTTCAAGATCGCA GTGTGTTTAGAATGGTGA	*bla*_OXA_	11

CTX-M-F CTX-M-R	CGCTTTGCGATGTGCAG ACCGCGATATCGTTGGT	*bla*_CTX-M_	11

CTX-M-F2 CTX-M-R2	CCAGAATAAGGAATCCCATG GCCGTCTAAGGCGATAAAC	whole *bla*_CTX-M_	11

CMY-F CMY-R	GACAGCCTCTTTCTCCACA TGGAACGAAGGCTACGTA	*bla*_CMY_	11

Plasmid-mediated quiniolone resistance			

qnrA-F qnrA-R	ATTTCTCACGCCAGGATTTG GATCGGCAAAGGTTAGGTCA	*qnrA*	19

qnrB-F qnrB-R	GATCGTGAAAGCCAGAAAGG ACGATGCCTGGTAGTTGTCC	*qnrB*	19

qnrS-F qnrS-R	ACGACATTCGTCAACTGCAA TAAATTGGCACCCTGTAGGC	*qnrS*	19

**Table 2:** Phenotypic and molecular characteristics of multidrug resistant Gram-negative bacteria isolates from flamingos

Isolate	Bacteria	Resistance phenotype	Integron/resistance gene (s)
AZ072-1-1	*E. coli*	AMP, SXT, CHL, TET, STR, NAL	Class 1 (*dfrA7*), *bla*_TEM-1_

AZ073-1-1	*E. coli*	AMP, SXT, STR, NAL, NOR, CIP	*qnrS*

AZ074-1-1	*E. cloacae*	AMP, STR, KAN, TET, CTX, CPD	*bla*_CTX-M-2_

AZ076-1-3	*C. youngae*	AMP, FOX, AMC, NAL^L^, CIP^L^	*qnrB*

AZ078-1-1	*E. coli*	AMP, SXT, STR, TET, CHL, NAL, AMC, ATM, FOX	Class 1 (*dfrA7*), *bla*_TEM-1_

AZ079-1-1	*E. coli*	AMP, STR, TET, KAN, CHL, SXT, NAL, ATM, CPD	Class 2 (*sat2-aadA1*), *bla*_TEM-1_

AZ0710-1-1	*Salmonella* *arizonae*	AMP, STR, TET, SXT, CHL, FOX, ATM, CPD, CTX, CRO	Class 1 (*dfrA7*)

AMP: ampicillin; AMC: amoxicillin-clavulanic acid; FOX: cefoxitin; CTX: cefotaxime; CPD: cefpodoxime; CRO: ceftriaxone; ATM: aztreonam; NAL: nalidixic acid; CIP: ciprofloxacin; NOR: norfloxacin; CHL: chloramphenicol; KAN: kanamycin; STR: streptomycin; TET: tetracycline; SXT: sulfamethoxazole-trimethoprim.

^L^: Low level of resistance.

Production of β-lactamases is the main mechanism of resistance to β-lactam antibiotics in Gram-negative bacteria. Many different β-lactamases have been described, but TEM-, SHV-, OXA-, CMY- and CTX-M-β-lactamases are the most predominant in Gram-negative bacteria [4]. The bacterial isolates were tested for TEM-, SHV-, CTX-M-, OXA- and CMY-β-lactamase-encoding genes by PCR using universal primers for the TEM, SHV, OXA, CTX-M and CMY families, as described previously (Table 1) [11]. PCR- and DNA-sequencing screening identified *bla*_TEM-1_, in three *E. coli *isolates (Table 2). It is well known that *bla*_TEM-1 _is a narrow-spectrum β-lactamase gene, which confers resistance against penicillins and first-generation cephalosporins [4]. TEM-β-lactamase has been previously detected in *E. coli *isolated from wild animals in Portugal [9] and from free-living Canada geese in Georgia and North Carolina (USA) and black-headed gulls in the Czech Republic [8,10]. In this study, an extended-spectrum β-lactamase (ESBL) encoding gene, *bla*_CTX-M-2_, was identified in a single isolate of *E. cloacae*. Notably, *bla*_CTX-M-2 _was previously identified in an *E. coli *strain isolated from masked palm civet at Hiroshima Zoological Park, Japan [11].

Plasmid-mediated quinolone resistance is of great concern since these resistance determinants are potentially spread among bacteria due to plasmid mobility. Until now, three main types of *qnr *gene have been identified: *qnrA*, *qnrB *and *qnrS *[18,19]. Multiplex PCR amplification was used for screening of plasmid-mediated quinolone resistance genes, *qnrA*, *qnrB *and *qnrS *as described previously (Table 1) [19]. PCR-screening and DNA-sequeencing results identified *qnrS *and *qnrB *in single isolates of *C. youngae *and *E. coli*, respectively (Table 2). In Japan, we previously identified six *qnrB *genes in *E. coli*, *K. pneumoniae, K. oxytoca, C. freundii, P. mirabilis *and *P. fluorescence *from zoo reptiles and falcons, and four *qnrS *genes in three isolates of *E. coli *and one isolate of *E. cloacae *from reptiles, red fox and snowy owl [11].

Contact with animals in zoological parks provides opportunities for entertainment and education regarding animals and animal husbandry; however, inadequate understanding of disease transmission and animal behavior can lead to infectious diseases and other health problems among visitors, especially children [20]. Regular training and education of animal care-takers and visitors by zoological parks are prerequisites to avoid exposure to these resistance genes. The National Association of State Public Health Veterinarians, Inc. (NASPHV) has provided standardized recommendations for public health officials, veterinarians, animal venue operators, animal exhibitors, visitors to animal venues and exhibits, and others concerned with disease-control and with minimizing risks associated with animals in public settings [20]. Also, we think that the most important measure to prevent introduction of antimicrobial resistance genes through exotic bacteria either to Japan or to any other country is to screen the imported animal flocks (either livestock or wildlife animals) for the presence of resistance genes in the quarantine. No flocks should be approved to enter the country if the screening results showed that they harbored of any clinically important resistance genes. In conclusion, theoretically, wild animals should never have been exposed to antibiotics; however, this study emphasizes the potential risk of imported zoo animals as a source and reservoir of MDR bacteria. It highlights their potential role in the spread of antimicrobial resistance genes from one country to another. Although, the source of resistance genes in the imported flamingos is unknown, the possibility remains that flamingos may become colonized with resistant bacteria during transit (in a possible storage facility in Tanzania, in vehicles, in the Netherlands, or during air transport). Finally, more research is needed to better understand the role of imported wild birds in transmission of resistance genes and its implications for public health.

## Competing interests

The authors declare that they have no competing interests.

## Authors' contributions

MS carried out the isolation and identification of bacteria, also, screening and identifying of resistance genes. AMA conceived of the study and participated in its design and experiments and drafted the manuscript. TS participated in the discussion on the study design and finalized the manuscript. AN, HW and YF made sampling and participated in the isolation and identification of bacteria. All authors read and approved the final manuscript.
